# Extremely high-dose insulin requirement in a diabetic patient with COVID-19: a case report

**DOI:** 10.1186/s12902-020-00632-2

**Published:** 2020-10-16

**Authors:** François R. Jornayvaz, Benjamin Assouline, Jérôme Pugin, Karim Gariani

**Affiliations:** 1 Division of Endocrinology, Diabetes, Nutrition and Therapeutic patient education, Geneva University Hospitals, Geneva, Switzerland; 2 Division of Intensive Care, Geneva University Hospitals, Geneva, Switzerland

**Keywords:** COVID-19, Insulin resistance, Type 2 diabetes, Case report

## Abstract

**Background:**

Detailed description of hyperglycemia management in diabetic patients infected with SARS-CoV-2 remain limited, although patients with diabetes show higher complication and mortality rate than patients without diabetes. Transient non-severe increased insulin requirement in patients hospitalized for medical conditions such as sepsis or myocardial infarction is a well-known phenomenon. However, extremely high-dose insulin requirement remains a very rarely reported entity. Here, we report the case of an extreme and transitory insulin requirement episode in a type 2 diabetic patient presenting an acute respiratory distress syndrome caused by SARS-CoV-2.

**Case presentation:**

A 57-year-old man resident in Geneva, Switzerland, previously known for type 2 diabetes for 3 years was admitted for an aggravation of his dyspnea. His type 2 diabetes was treated only with metformin and his latest Hb1Ac was 6.1%. Chest CT SCAN showed a bilateral multilobar ground-glass opacification. Twenty-four hours after his admission he presented a worsening of dyspnea and severe hypoxemia requiring a transfer to the intensive care unit rapidly followed by oro-tracheal intubation for mechanical ventilation support. A bronchoalveolar lavage was performed and test of SARS-CoV-2 by RT-qPCR assay was positive. At day 3, he presented a rapidly progressive insulin requirement at a rate of up to 50 units/hour intravenous insulin aspart. Despite the high insulin doses, he maintained an elevated plasma glucose level at 270 mg/dL on average. His extremely high-dose insulin requirement “resolved” at day 9, and the insulin infusion rate was rapidly reduced.

**Conclusions:**

This case may reflect a specific and profound impact of SARS-CoV-2 on metabolic homeostasis, in particular in diabetic patients that appear more prone to complications of COVID-19 infection. Yet, the mechanisms behind this remain to be elucidated. The optimal management of hyperglycemia of diabetic patients infected with SARS-CoV-2 has yet not be defined, however insulin remain the mainstay of treatment approach. Report of extreme dysregulation of chronic conditions such as diabetes in patients with COVID-19 may help clinicians to better take care of patients during the pandemic of SARS-CoV-2.

To the best of our knowledge this is the first description of extremely high-dose insulin requirement in patient with COVID-19.

## Background

Since December 2019, an outbreak of coronavirus disease, officially named by the World Health Organization as COVID-19, appeared in Wuhan, Hubei Province, China. Diabetic patients infected with SARS-CoV-2 appear more prone to require hospitalization and admission to intensive care unit.

Transient non-severe increased insulin requirement in patients hospitalized for various medical conditions such as sepsis or myocardial infarction is a well-known phenomenon with insulin doses usually not exceeding 5–10 Units/hours [1]. However, extremely high-dose insulin requirement remains a very rare reported entity. Here, we report the case of an extreme and transitory high-dose insulin requirement episode in a diabetic patient presenting an acute respiratory distress syndrome (ARDS) caused by SARS-CoV-2.

## Case presentation

A 57-year-old man previously known for type 2 diabetes (T2D) for 3 years, asthma and obstructive sleep apnea was admitted for an aggravation of his dyspnea. Bodyweight was 99 kg, height was 172 cm and body mass index was 33.5 kg/m^2^.

His T2D was treated only with metformin 500 mg twice daily and his latest Hb1Ac was 6.1%. The patient was not on other regular treatment. In the past 3 weeks, he presented a dry cough that was initially managed with amoxicillin/clavulanic acid and clarithromycin for 5 days. At admission, he presented a normal leucocyte count at 4.3 G/l with lymphopenia at 0.39 G/l count and an elevated CRP at 58 mg/l [2, 3]. Chest CT SCAN showed a bilateral multilobar ground-glass opacification. A nasopharyngeal swab test of SARS-CoV-2 by RT-qPCR was performed the same day in another medical facility and the result was pending.

Twenty-four hours after his admission he presented a worsening of dyspnea, with tachypnea and severe hypoxemia requiring a transfer to the intensive care unit rapidly followed by oro-tracheal intubation for mechanical ventilation support. A vasopressor infusion (norepinephrine) was started. A bronchoalveolar lavage was performed and test of SARS-CoV-2 by RT-qPCR assay was positive. At day 3, he presented a rapidly progressive insulin requirement at a rate of up to 50 units/hour iv of insulin aspart (Table 1). Despite the high insulin doses, he maintained an elevated plasma glucose level at 270 mg/dL on average.

**Table 1:** clinical and biological parameters during intensive care unit stay

	Day 1	Day 2	Day 3	Day 4	Day 5	Day 6	Day 7	Day 8	Day 9	Day 10	Day 11	Day 12	Day 13	Day 14	Day 15
Total insulin requirement per day (UI)	10 iv	120 iv	384 iv	633 iv	1035 iv	583 iv	387 iv	554 iv	348 iv	78 iv	84 iv	85 iv	65 iv	60 sc	60 sc
CRP (mg/l)	179	275	300	251	192	199	169	98	43	72	101	95	79	–	22
PCT (μg/l)	0.56	0.86	0.74	0.7	–	0.93	–	0.57	0.49	0.77	–	–	–	0.31	–
Nutrition	Enteral	Enteral	Enteral	Enteral	Enteral	Enteral	Enteral	Enteral	Enteral	Enteral	Enteral	Enteral	Enteral	Enteral	Enteral
Carbohydrates (g)	91.5	91.5	141.55	141.5	141.5	141.5	141.5	141.5	141.5	183	183	183	183	183	183
Corticosteroids	–	–	–	–	–	Hydrocortisone 50 mg 3x/d	Hydrocortisone 50 mg 3x/d	Hydrocortisone 50 mg 3x/d	–	–	–	–	–	–	–
vasopressor infusion (norepinephrine) (μg/kg/min)	0.22	0.28	0.28	0.21	0.20	0.16	0.04	0.02	No	No	No	No	No	No	No
Plasma glucose level (mg/dl)	106	227	232	290	274	260	243	234	155	137	216	229	155	157	122
Clinical evolution	Admission at Intensive care unit and oro-tracheal intubation												Extubation	Transfer to the intermediate care unit	

Table Legend: Evolution of insulin requirement and inflammatory and clinical parameters during intensive care unit stay. *CRP* C-reactive protein, *PCT* procalcitonin, *iv* intravenous, *sc* subcutaneous

His extremely high-dose insulin requirement “resolved” at day 9, and the insulin infusion rate was rapidly reduced, avoiding hypoglycemia (Table 2). He was extubated at day 13 and discharged to an intermediate care unit as a step down approach to pursue a close monitoring and regular respiratory care including continuous positive airway pressure (CPAP), and 2 days later he was transferred to regular hospital room due a positive clinical evolution. His insulin treatment was switched to subcutaneous neutral protamine Hagedorn (NPH) insulin at a total dose of 60 UI per day.

**Table 2:** Laboratory values at intravenous insulin initiation and withdrawal

Variables	At intravenous insulin initiation	At intravenous insulin withdrawal
Leukocytes count (10^− 9^/l)	11.4	6.0
Lymphocytes count (10^− 9^/l)	0.52	1.22
**C-reactive protein** (mg/l)	179	14
Procalcitonin (μg/l)	0.56	N.A.
Creatinine (μmol/l)	80	106
Urea (mmol/l)	2.6	6.4
Aspartate aminotransferase (U/liter)	48	44
Alanine aminotransferase (U/liter)	37	25
Bilirubin conjugate (μmol/l)	11	7
Albumin (g/l)	20	36
Potassium (mmol/l)	3.6	3.8
Sodium (mmol/l)	135	138
Magnesium (mmol/l)	0.78	0.8
Phosphate (mmol/l)	0.86	1.28
Chloride (mmol/l)	98	102

*N.A*. non available

## Discussion and conclusions

This case could reflect a potential severe impact of COVID-19 infection on metabolic homeostasis in a sub-group of patients, in particular diabetic individuals that appear more prone to complications of COVID-19 infection. Yet, the mechanisms behind this remain to be elucidated. No previous report has been made in COVID-19 infection of extremely high-dose insulin requirement. Available data remain limited to assess if COVID-19 infections are more prone to induce metabolic disturbances than SARS-CoV, but previous observations with SARS in 2003 showed that both hyperglycemia and history of diabetes were independent predictors of severe respiratory symptoms and death in SARS-CoV patients, as observed with COVID-19 infection [4–7].

The metabolic response to sepsis encompasses several phenomenons that can contribute to hyperglycemia such as the activation of the hypothalamic-pituitary-adrenal axis, leading to the secretion of cortisol, growth hormone and glucagon resulting in an increased level of glucose. In addition, several mediators of the systemic inflammatory response such as TNF-α or IL-1 may cause hepatic and muscle insulin resistance by disturbing insulin receptor signaling [8]. Interestingly COVID-19 infected patients often present normal or low leucocytes counts and lymphopenia as in our patient [9]. Furthermore, endogenous and exogenous catecholamine used as vasopressors have been reported to increase glucose levels by stimulating glycogenolysis, gluconeogenesis, inhibiting glycogen synthesis and stimulating the release of growth hormone and glucagon [10]. Moreover, a study showed that SARS-CoV may cause diabetes by ACE2-dependent damage of pancreatic islet cells [11]. and similar observation have been made in patients infected with SARS-CoV2, suggesting that this mechanism could be one the factors contributing to metabolism homeostasis disturbance leading to new-onset diabetes and acute hyperglycemia in COVID-19 infection [12].

In the present case, we could hypothesize that COVID-19 infection has led to a high insulin resistance state through several mechanisms including a large release of cytokines named cytokine storm as described in several reports. In addition, catecholamine administration may have exacerbated this state [13]. Furthermore, T2D is often associated with insulin-resistance due to a low-grade inflammatory state and abnormal plasma oxidative stress that can be exacerbated during an infection episode, leading to intense insulin resistance as presented by our patient [8].

Observational studies are required to better elucidate the impact of COVID-19 infection on glucose metabolism, in particular in diabetic patients, by several approaches such as comparing insulin requirement between patients with COVID-19 infection and patients with other causes of septic shock and ARDS to eventually confirm a potential disproportionate insulin requirement during COVID-19 infection.

## Data Availability

The data of this study may be available on reasonable request to the corresponding author.

## Abbreviations

ARDS: Acute respiratory distress syndrome
CPAP: Continuous positive airway pressure
NPH: Neutral protamine Hagedorn
T2D: Type 2 diabetes
